# Protocol for spatially restricted CreERT2-mediated recombination in mouse brain-infiltrating B cells using intracerebral 4-hydroxy-tamoxifen

**DOI:** 10.1016/j.xpro.2026.104856

**Published:** 2026-09-21

**Authors:** Mika Schneider, Georgios Perdikaris, Tobias Derfuss, Nicholas S.R. Sanderson

**Affiliations:** 1 Department of Biomedicine, University Hospital Basel and University of Basel, Basel, Switzerland; 2 Multiple Sclerosis Centre and Research Center for Clinical Neuroimmunology and Neuroscience (RC2NB), University Hospital Basel and University of Basel, Basel, Switzerland; 3 Department of Neurology, University Hospital Basel, Basel, Switzerland

**Keywords:** Immunology, Neuroscience, Systems biology

## Abstract

Tamoxifen-dependent, cre-recombinase-mediated techniques enable precise temporal control of gene expression, but do not always provide sufficient anatomical control. Here, we present a protocol to restrict gene expression using local injection of 4-hydroxy-tamoxifen in bone wax. As an example, we describe the induction of the viral protein LMP1 and the fluorescent protein tdTomato only in brain-infiltrating B cells. We describe steps for intracerebral virus injection and 4-hydroxy-tamoxifen administration in mice.

For complete details on the use and execution of this protocol, please refer to Kim et al.^1^

## Highlights

• Steps for intracerebral virus injection
• Steps for mixing bone wax with 4-hydroxy-tamoxifen
• Steps for intracerebral 4-hydroxy-tamoxifen administration

## Abstract (editor highlights)

Publisher’s note: Undertaking any experimental protocol requires adherence to local institutional guidelines for laboratory safety and ethics.

## Graphical abstract

**Figure:**
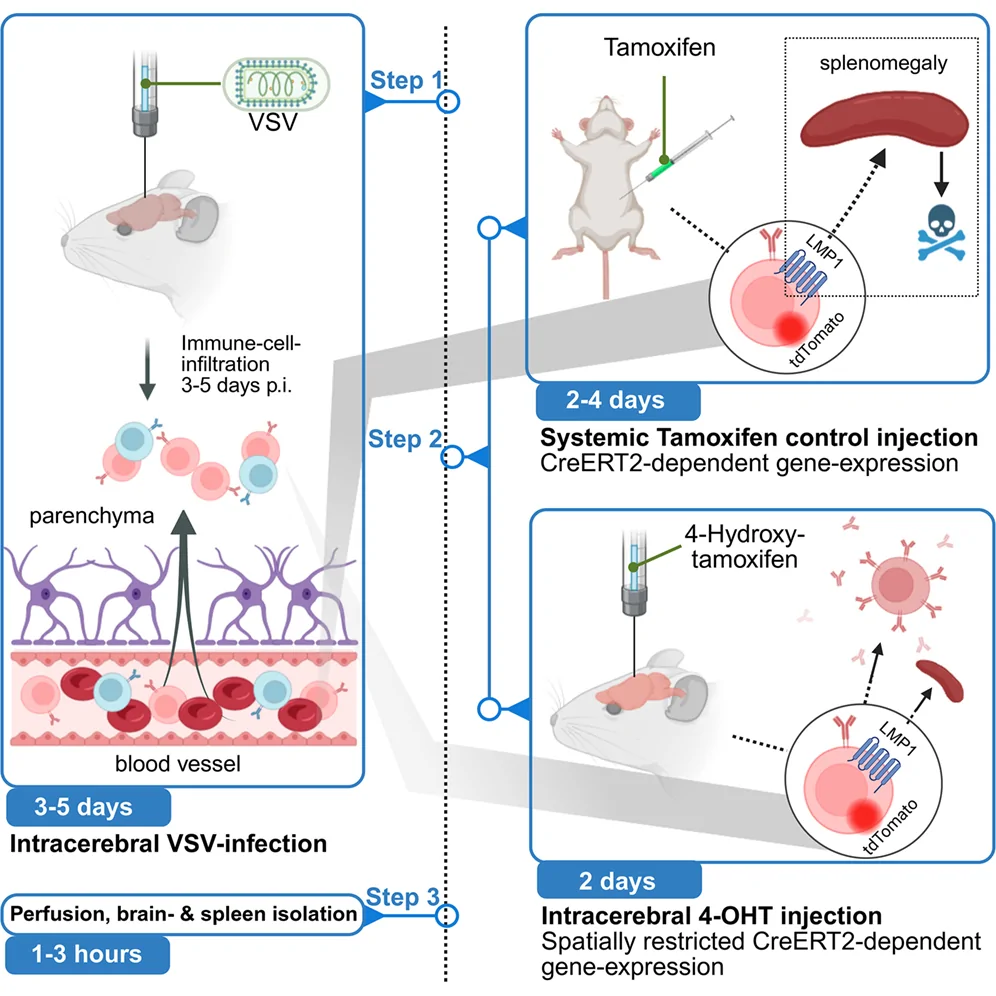

## Before you begin

The B-cell-tropic Epstein–Barr virus (EBV) markedly increases the risk of various autoimmune diseases, including multiple sclerosis.^2^^,^^3^^,^^4^

We have previously demonstrated that myelin-reactive B cells expressing the EBV gene latent membrane protein 1 (LMP1) can evade activation-induced cell death (AICD), thereby enabling autoantibody-driven demyelination in the brain.^1^ We found that naïve, autoreactive B cells undergo cell death upon antigen encounter following entry into the mouse brain, whereas LMP1 expression promotes their survival, proliferation, and capacity to induce demyelination.^1^ Because EBV does not infect non-human B cells, other methods are required to study the action of this gene in mouse models. Transgenic mice ae available that express LMP1 in B cells after tamoxifen injection, but expression of the protein by circulating B cells rapidly leads to massive B cell proliferation and ultimately death.^1^ Here, we present a protocol that enables anatomically restricted recombination of genes in B cells using a tamoxifen-dependent CreERT2 system. In the system we describe here, this corresponds to the recombination of tdTomato and LMP1 proteins exclusively in those B cells that have infiltrated the brains of transgenic mice in response to Vesicular Stomatitis Virus (VSV) infection, while leaving peripheral and circulating B cells unaffected. We describe the steps for intracerebral VSV infection by stereotactic injection, and outline the procedure for local 4-hydroxy-tamoxifen administration. We have used this approach with male and female animals between the ages of 6 to 24 weeks, at weights between 20 and 35 grams. Although this protocol is optimized for intracranial application, the principle of localized CreER activation may be adaptable to other tissues following appropriate optimization of local delivery methods. In principle, any genotype in which systemic tamoxifen is effective ought to be amenable to this approach.

### Innovation

The Cre-lox system has fundamentally advanced our ability to investigate gene function by enabling cell type-specific gene expression using targeted promoters.^5^ The subsequent development of tamoxifen-inducible CreERT2 systems expanded this capability by introducing precise temporal control of gene recombination,^6^ making these models extremely powerful.

Building on these established technologies, our model introduces an additional level of specificity by enabling anatomically restricted gene recombination. By injecting the active tamoxifen metabolite 4-hydroxy-tamoxifen in a bone wax substrate to restrict its diffusion, we achieve localized induction of Cre activity. In contrast to conventional intraperitoneal tamoxifen administration, which induces recombination in defined cell types across the entire organism, our approach confines recombination to a small, anatomically restricted subset of cells within a given tissue. Tamoxifen itself is a prodrug that requires hepatic metabolism to generate the active compound, 4-hydroxy-tamoxifen (4-OHT).^7^ Consequently, for anatomically targeted recombination, direct application of the active metabolite is necessary.

This innovation provides a critical advantage for studying genes with potentially deleterious systemic effects. In our experimental setting, it enables the investigation of the impact of Epstein–Barr viral LMP1 on B cells in the central nervous system while avoiding the risk of systemic toxicity and premature mortality in the animal model.^1^

### Institutional permissions

Studies involving live animals were reviewed and authorized by the animal research office of the cantonal veterinary department of Basel, and the tri-cantonal Animal Research Commission of Basel and Aargau.

## Key resources table

**Table:** 

REAGENT or RESOURCE	SOURCE	IDENTIFIER
**Bacterial and virus strains**		

VSV∗ΔG (5000 TU/ul)	Hoffmann et al.^8^	N/A

**Experimental models: Organisms/strains**		

Mouse: BALB/C-GT(ROSA)26sor^tm10(LMP1)Rsky/^J	The Jackson Laboratory	RRID:IMSR_JAX:019120
Mouse: B6.C-CD79A^tm3(CRE/ERT2)Reth/^EhobJ	The Jackson Laboratory	RRID:IMSR_JAX: 033026
Mouse: B6.CG-GT(ROSA)26SOR^TM14(CAG−TDTOMATO)HZE/J^	The Jackson Laboratory	RRID:IMSR_JAX: 007914

**Chemicals, peptides, and recombinant proteins**		

Bone wax	B Braun	Cat# 1029754
Tamoxifen	Sigma-Aldrich	Cat#T5648; CAS-No: 10540-29-1
4-hydroxy-tamoxifen	Sigma-Aldrich	Cat#SML1666. CAS-No: 68392-35-8
Corn oil	Sigma-Aldrich	Cat#C8267; CAS-No: 8001-30-7
Attane Isoflurane ad us. Vet.	Piramal Pharma	Cat#AP/DRUGS/220/96
Lidocaine 2%	Streuli Pharma	Cat#AE16005/000.0
Bupivacaine 5 mg/mL	Sintetica	Cat#6811006
Rimadyl ad us. Vet. Carprofenum	Zoetis Schweiz GmbH	Cat#40039886
Betadine	Mundi pharma	Cat#14670-1805
Lacrinorm Carbomerum	Bausch + Lomb	Cat#48038T574
NaCl 0.9% solution	B. Braun Medical AG	Cat#395202

**Antibodies**		

DAPI	Sigma-Aldrich	Cat#D9542
Alexa Fluor 647 anti-mouse CD45R (B220)	BioLegend	Cat#103226; RRID:AB_389330
Brilliant Violet 510 anti-mouse CD19	BioLegend	Cat#115545; RRID:AB_2562136
Alexa Fluor 647 anti-mouse CD45	BioLegend	Cat#103124; RRID:AB_493533

**Other**		

Dafilon 5/0 DS19	B. Braun	Cat#C0932191
Sterican safety 22G, 0.70 × 30 mm, black	B Braun	Cat# 4657624B
Sterican safety 25G, 0.50 × 16 mm, orange	B Braun	Cat# 4657853B
Carbon steel surgical blade	Swann-Morton	Cat# 0201
Hamilton syringe	Hamilton	Cat# 80330
Omnifix-F Syringe, 1 mL, LS	B Braun	Cat# 9161406V
RightTemp. Jr., far infrared warming pad	Kent Scientific	Cat# RTJR

## Step-by-step method details

The sequence of procedures is shown schematically in Figure 1.

**Figure 1:**
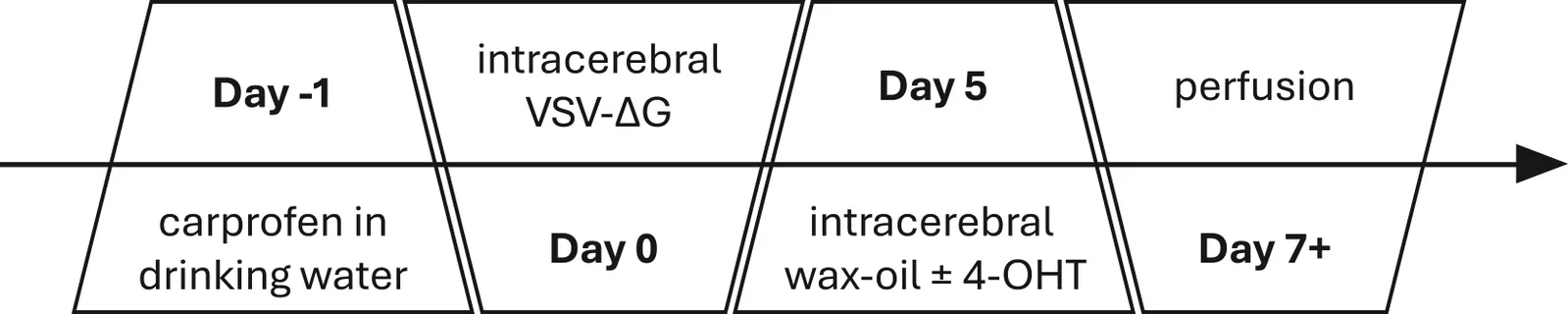
Experimental timeline Carprofen provided in the drinking water from day −1, followed by intracerebral VSV-ΔG injection on day 0, intracerebral wax-oil ± 4-OHT injection on day 5, and perfusion on day 7 or later according to experimental endpoint.

### Intracranial injection of VSV-ΔG into the striatum

**Timing: 1.5 h (hands-on) + 5 days of post- VSV∗ΔG- infection phase**

The following steps are performed on day 0 under aseptic conditions in both experimental and control mice anesthetized with isoflurane and given suitable analgesics. starting, thaw a 30 μL aliquot of VSV∗ΔG^8^ (5000 transducing units per microliter) and keep on ice until use. Any CreRT2 mouse line can be used; the following example uses WT tam(LMP1 tomato) mice (for details of the crossing scheme please refer to Kim et al.^1^), in which active Cre mediates excision of STOP codons from two transgenes – the fluorescent protein tdTomato, and the Epstein-Barr viral protein LMP1.

1. Pre-operative preparation (10 min).
  a. The day before surgery, add Rimadyl (carprofen) to normal drinking water (not acidified) to provide prophylactic analgesia (0.067 mg/mL), and continue carprofen for 3 days after surgery.
  b. Check all connections of the isoflurane anesthesia system and confirm correct function prior to experimental start.
  c. Sterilize surgical instruments in a bead sterilizer according to institutional guidelines (instruments shown in Figure 2A, 1–12).

**Figure 2:**
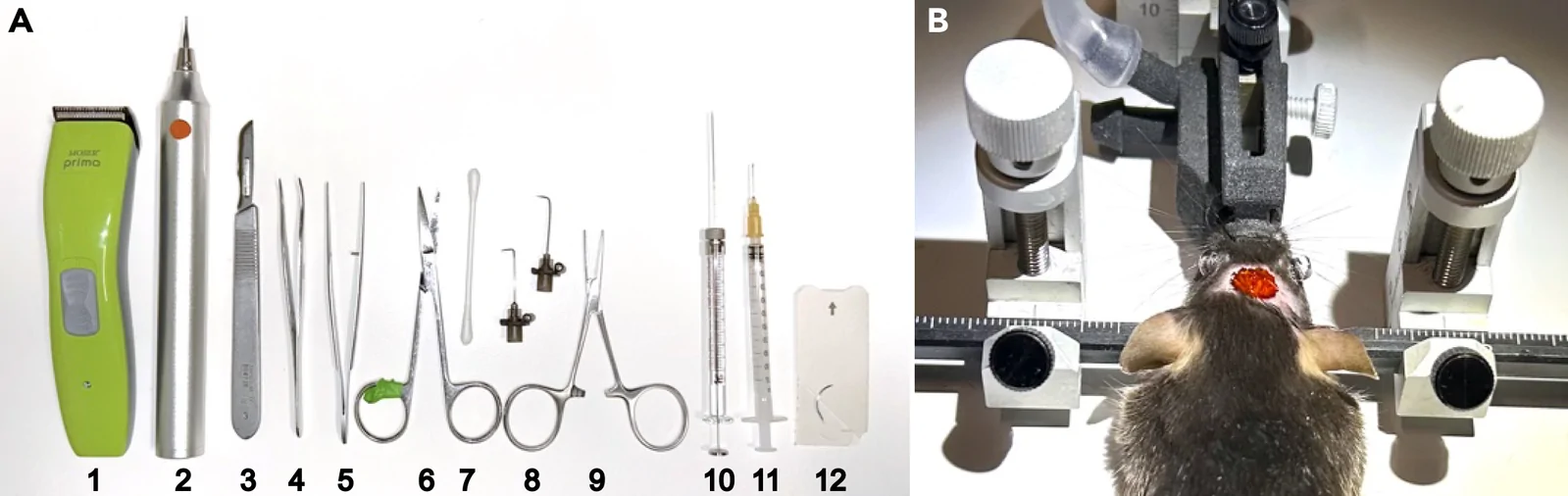
Preoperative setup for mouse surgery (A) Instruments and tools from left to right: (1) electric hair clipper; (2) electric drill; (3) scalpel handle with blade; (4) fine tissue forceps; (5) thumb forceps; (6) operating scissors; (7) cotton swabs; (8) bent 22G needle; (9) mosquito hemostat; (10) Hamilton syringe 10 μL; (11) 1 mL syringe with 25G needle; (12) suture with curved surgical needle. (B) Anesthetized mouse positioned in a stereotactic frame following ocular protection, scalp shaving and antiseptic preparation.
  d. Maintain the mouse’s body temperature throughout the surgical procedure using a temperature-controlled, pre-warmed heating pad.

***Note:*** Post-surgical analgesia with carprofen in drinking water is only effective if the animals continue to drink normally. This should be monitored by measuring water consumption before and after surgery. Other analgesic approaches, such as injection, or longer-acting agents should be discussed with local veterinary experts, and may be mandated by ethical oversight authorities.

2. Induction and maintenance of anesthesia and positioning in the stereotactic frame (10 min).
  a. Weigh each mouse and record the body weight.
  b. Place the mouse in the induction chamber and anesthetize with 3%–5% isoflurane in oxygen until the animal reached deep surgical plane of anesthesia (no response to toe pinch, slow stable breathing).
  c. Reduce isoflurane concentration to 1.5%–3% (in O_2_) for maintenance and confirm that respiration is slow and stable, not rapid.
  d. Transfer the anesthetized mouse with the heating pad to the stereotactic frame and position the nose in the anesthesia nose cone to maintain isoflurane anesthesia.
  e. Fix the head firmly in the stereotactic frame using the metal ear bars and nose clamp so that no movement is possible (shown in Figure 2B) taking care to adjust the positioning delicately to avoid damaging sensitive structures such as the ears, nose, and teeth.
3. Preparation of the surgical field (shown in Figure 2B) (10 min).
  a. Shave the scalp over the surgical area with electric clippers (ensure complete hair removal over the surgical field, avoiding skin irritation).
  b. Apply eye gel (Lacrinorm) to both eyes to prevent corneal drying during anesthesia.
  c. Inject 30–50 μL of a mixture of bupivacaine and lidocaine (0.25% and 0.5% w/v respectively) in 0.9% NaCl subcutaneously along the planned incision line for local analgesia.
    ***Note:*** Sufficient solution should be injected to cause a visible elevation of the scalp.
  d. Disinfect the shaved area thoroughly with betadine (povidone-iodine) and allow a contact time of approximately 5–10 min before incision.
    **CRITICAL:** 5–10 min must elapse between injection of local analgesics and the incision of the scalp to allow the drugs to act. The combination of drugs is used because lidocaine acts in 5–10 min but is not effective for long enough to guarantee effective analgesia for the duration of the surgery, while bupivacaine takes longer to act but is effective for longer (Durst et al., 2021).^9^
    Exposure of the skull (10 min).
  e. Using a sterile scalpel blade, perform a mid-sagittal skin incision of approximately 1.5–2 cm to expose the skull (shown in Figure 3A).

**Figure 3:**
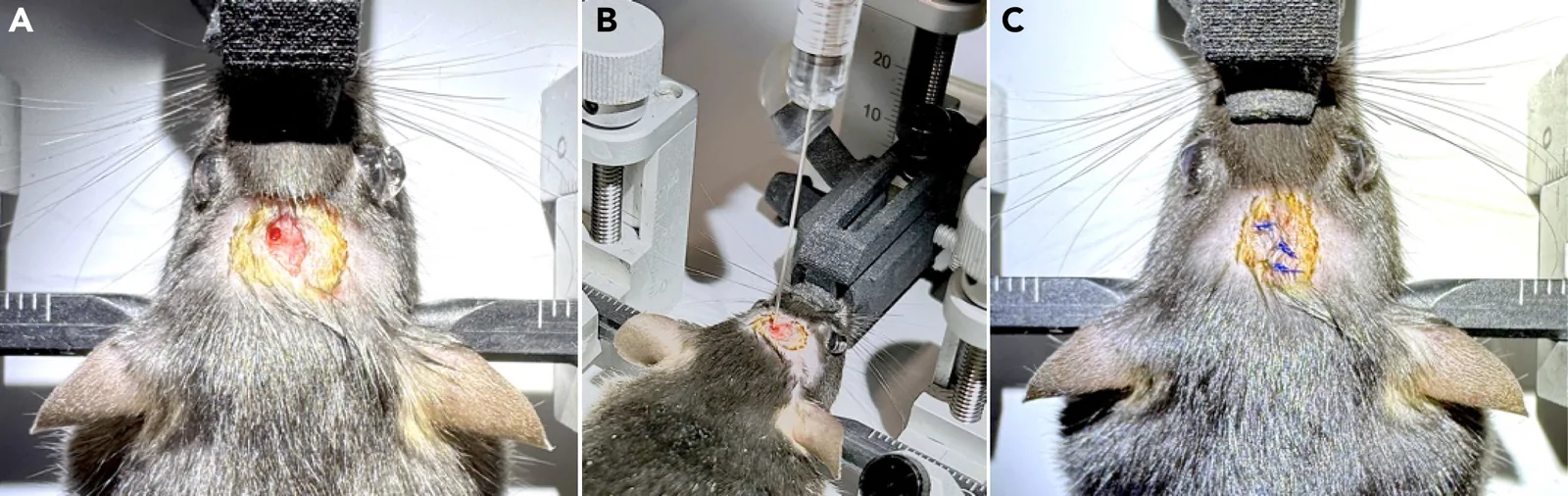
Stereotactic intracranial injection procedure in mice (A) Cranial skin incision and burr hole preparation. (B) Stereotactic positioning and insertion of a Hamilton syringe for intrastriatal injection. (C) Wound closure of skin with suture after completion of procedure.
  f. Gently remove connective tissue from the skull surface using a bent hypodermic needle (Figure 2A (8)) until bregma is clearly visible.
  g. Keep the surgical field moist as needed with sterile saline and ensure clear visualization of bregma for accurate coordinate setting.
4. Determination of injection coordinates and drilling (10 min) (troubleshooting 1).
  a. Using the stereotactic frame, position the needle tip or a reference point over bregma and zero the coordinates.
  b. Move to the target coordinates for striatal injection: 1.5–2 mm lateral and 1 mm rostral relative to bregma.
  c. At the target position drill a burr hole of approximately 0.8–1 mm diameter, taking care not to damage the dura, using a 0.8 mm drill bit (shown in Figure 3A).
  d. In case of bleeding during drilling, stop the procedure and achieve hemostasis before proceeding.
  e. Incise the dura at the injection site with a sterile hypodermic needle.

***Note:*** Stereotactic coordinates must be adapted to the target structure, and the age and genetic background of the mouse. An alternative to using separate animals for controls is a control injection at the same locus contralaterally.

5. Virus injection (10–15 min).
  a. Load the Hamilton syringe with 5000 transducing units per μL of VSV∗ΔG according to biosafety regulations, avoiding air bubbles.
  b. Position the syringe over the burr hole using the stereotactic frame.
  c. Lower the needle at 1–2 mm per minute to a depth of 3 mm below the dura, then immediately withdraw the needle 0.5 mm to reach a final injection depth of 2.5 mm (shown in Figure 3B).
  d. Inject 1 μL of VSV∗ΔG over 100 s (0.1 μL every 10 s) by manually controlled injection speed, or an automated pump if available.
    ***Note:*** Injecting virus too fast will result in reflux up the needle track and peripheral immunization.
  e. After the injection is complete, keep the needle in place for 3 min to allow diffusion of the virus into the surrounding tissue.
6. Needle withdrawal and wound closure (5 min).
  a. Withdraw the needle slowly in 0.5 mm steps every 30 s until the needle is completely out of the brain.
  b. Inspect the burr hole and surrounding tissue for bleeding and manage as needed.
  c. Close the skin incision with sutures (Dafilon 5/0, DS19), to achieve adequate wound closure (shown in Figure 3C).
7. Post-operative recovery (20–30 min).
  a. Carefully release the animal from the stereotactic frame and transfer it to a pre-warmed recovery cage under a heat lamp.
  b. Cover the mouse lightly with tissue paper to reduce heat loss while ensuring that the animal has sufficient space to move away from the direct heat source.
  c. Monitor the mouse continuously until it regains consciousness and shows spontaneous movement.
  d. Once fully awake, return the mouse to its home cage with access to carprofen-containing drinking water (for up to 3 days post-surgery) and continue routine post-operative monitoring according to institutional guidelines.

### Preparation of Hamilton syringe with 4-hydroxy-tamoxifen-Corn oil-Bone wax mixture

**Timing: 1 h (hands-on)**

The following steps are performed on day 5 under sterile conditions. Before starting, heat the water bath to 80°C.

8. Preparation of bone wax - corn oil mixture (30 min).
  a. Cut off the required amount (60–75 mg yields enough for 6 mice) of bone wax from the block.
  b. Transfer the bone wax into a 500 μL Eppendorf tube.
  c. Add two volumes (150 μL for 75 mg of wax) of corn oil to the tube.
  d. Place the tube in a water bath until both components become fully liquefied (15 min).
  e. Mix thoroughly with multiple strokes of a 1 mL syringe connected to an 18G needle to obtain a homogeneous solution.

**CRITICAL:** The mixture of oil and wax should achieve a viscosity that can be easily extruded from the needle of the syringe. If it is too viscous, the oil content can be increased.

9. Preparation of control injection mixture (without 4-OHT) (10 min) (troubleshooting 2).
  a. Add 50 μL of ethanol (100%) into a new 500 μL Eppendorf tube.
  b. Add 30 μL of the mixture prepared in step 1
  c. Keep the tube in the waterbath and mix thoroughly to obtain a homogeneous solution. During this time, most of the ethanol vehicle should evaporate.
  d. Slowly aspirate the mixture prepared in step 1 into the Hamilton syringe and let it cool to 22°C–26°C.
10. Preparation of experimental injection mixture (with 4-OHT) (30 min) (troubleshooting 2).
  a. Add 50 μL of 4-hydroxy-tamoxifen (20 mg/mL in ethanol) into a new 500 μL Eppendorf tube.
  b. Add 30 μL of the mixture prepared in step 1 (12.5 mg/mL 4-OHT final concentration).
  c. Keep the tube in the waterbath and mix thoroughly to obtain a homogeneous solution. During this time, most of the ethanol vehicle should evaporate.
  d. Slowly draw the mixture into the Hamilton syringe and let cool to 22°C–26°C (20 min) protected from light to prevent photodegradation of 4-OHT.

**CRITICAL:** Make sure not to include air bubbles in the syringe

### Intracranial injection of 4-hydroxy-tamoxifen into the striatum

**Timing: 1.5 h (hands-on) + 2 days post- 4-OHT -injection phase**

The following steps are performed on day 5 under aseptic conditions in both experimental and control mice anesthetized with isoflurane and given suitable analgesics. Perform the “Intracranial injection of 4-hydroxy-tamoxifen into the striatum” exactly as described in Steps 1–8 of the “intracranial injection of VSV-ΔG into the striatum” major section, with the following modification:

Before performing a mid-saggital skin incision in step 4, remove sutures from the first surgery with scissors.

Do not repeat Step 5c and 5d, as there should already be a burr hole at the target position from the first surgery, which will also be used for this injection.

Replace Steps 6 a-e with the following.

11. 4-hydroxy-tamoxifen (or control mixture) injection (10–15 min).
  a. Position the pre-loaded syringe (as described in preparation section) over the burr hole using the stereotactic frame.
  b. Lower the needle to a depth of 3 mm below the dura, then immediately withdraw the needle 0.5 mm to reach a final injection depth of 2.5 mm (shown in Figure 3B).
  c. Inject 1 μL of 4-hydroxy-tamoxifen enriched bone wax - corn oil (=12.5 μg 4-OHT) at 22°C–26°C over 100 s (0.1 μL every 10 s) by manually controlled injection speed, or an automated pump if available.
  d. After the injection is complete, keep the needle in place for 3 min.

### Perfusion and organ analysis by histology and flow cytometry

Detailed description for perfusion, fixation, brain-, cervical lymph node- and spleen isolation, tissue embedding in TopVision low melting point agarose gel, tissue sectioning with the vibratome, immunofluorescence labeling and spleen preparation for flow cytometry analysis can be found in Kim et al., 2026.^1^

## Expected outcomes

Depending on the system, transgene expression should be visible in the target cells in the vicinity of the injection within 2–3 days, as shown in Figure 4A. Because of strong autofluorescence at the site of the injection (Figure 4B), it is important to have an additional marker to identify bona fide transgene expression. In the case of B cells, this is conveniently achieved with immunolabeling of a reliable B cell marker such as B220 – it is then easy to distinguish between autofluorescent debris, which is randomly distributed, and the transgenic tdTomato, whose location tightly follows the B cell marker.

**Figure 4:**
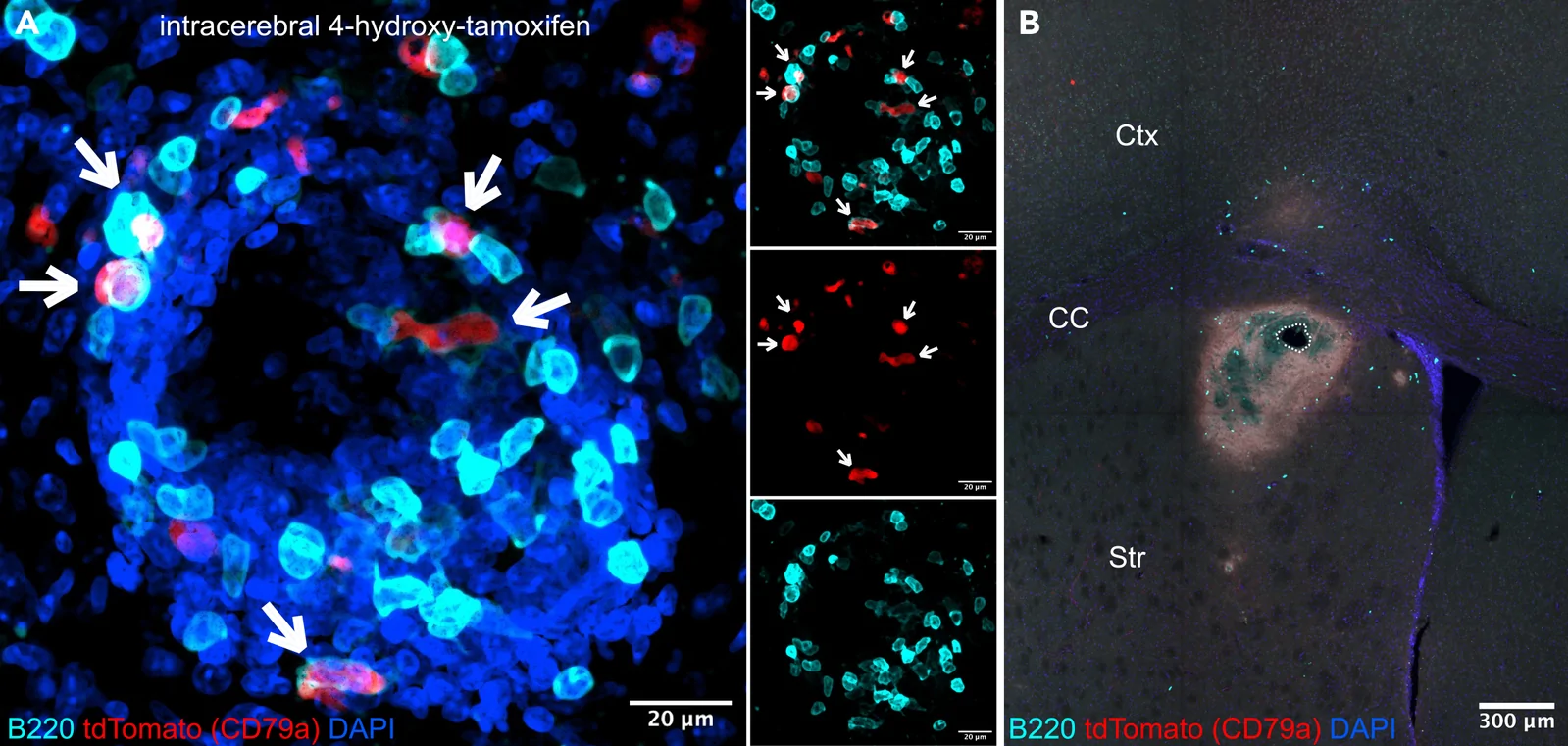
Intracerebral tamoxifen induces recombination *in situ* (A) Confocal micrographs of B cells (B220 in cyan), some of which express tdTomato (red) - CreERT2 expressed from the CD79a B cell promoter, under the influence of 4-OHT mediates excision of a STOP codon from the tdTomato gene in the ROSA26 locus. DAPI in blue marks cell nuclei. Scale bar in panels = 20 μm (60× magnified). White arrows indicate B220+, tdTomato+ B cells after intracerebral wax injection with 4-OHT. Smaller panel on right show single and double channels. (B) Lower powered 10× image showing the injection site in the context of the striatum. Cyan shows immunofluorescent labeling of the B220 (B cells). Dark blue = DAPI. Red includes the tdTomato signal from the recombined B cells, but at this magnification this signal cannot be distinguished from autofluorescence (see 4a). The broken white line surrounds the area of wax deposition (wax is usually displaced from the sections during cutting, leaving a lacuna. (CC = corpus callosum, Ctx = cortex, Str = striatum). Scale bar = 300 μm.

In addition, tight restriction of recombination to the target region can be confirmed with suitable observations of non-target tissues. For example, the flow cytometric analysis of the splenic B cells (shown in Figure 5A) and histological analysis of the deep cervical lymph nodes (shown in Figure 5B) allow assessment of potential peripheral effects of intracerebral 4-hydroxy-tamoxifen administration. Note however, that many nominally cre-dependent transgenic systems show some measure of “leakage” – in our system between 0.01 and 0.1% of splenic B cells express tdTomato without any tamoxifen administration. Using this protocol, no evidence of additional peripheral recombination was detected, indicating that recombination remains restricted to the site of injection under the described conditions.

**Figure 5:**
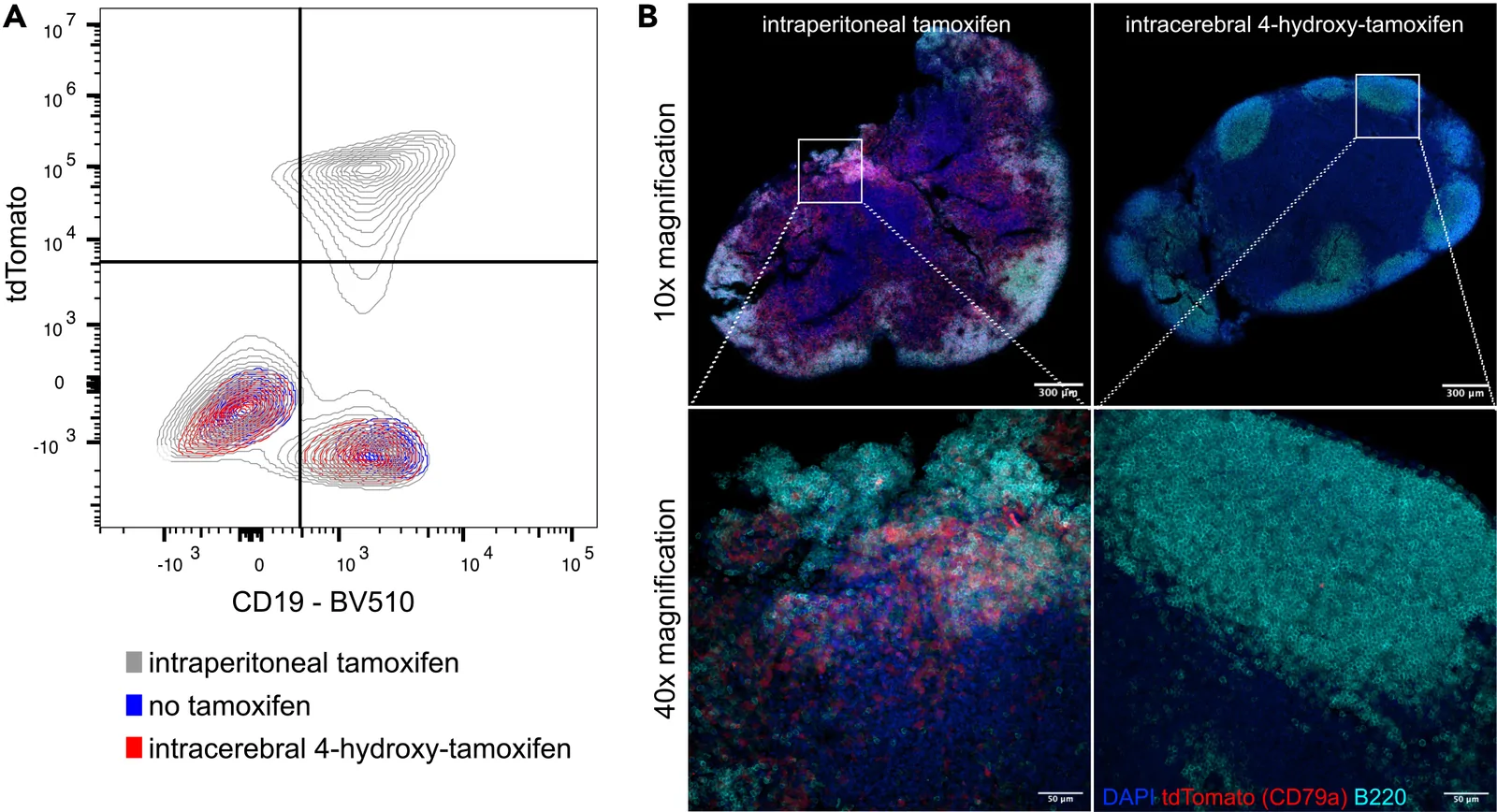
Intracerebral tamoxifen does not induce peripheral recombination (A) Flow cytometric analysis of splenic B cells (CD45+CD19+). Expression of tdTomato in B cells only in intraperitoneal tamoxifen recipients (grey), but not in intracerebral treated (red) or untreated animals (blue). Vertical axis: tdTomato; horizontal axis: CD19. (B) Confocal micrographs of deep cervical lymph node B cells. B220 in cyan, tdTomato (B cells) in red (CreERT2 expressed from the CD79a B cell promoter mediates excision of a STOP codon from the tdTomato gene in the ROSA26 locus) and DAPI in blue. Scale bar in panels = 300 μm (10× magnified) or 50 μm (40× magnified). Left panels show B220+ tdTomato+ B cells after intraperitoneal tamoxifen, right panels show B220+tdTomato-after intracerebral 4-hydroxy-tamoxifen.

## Limitations

Achieving efficient recombination of tamoxifen-dependent proteins in B cells migrating into the brain is subject to both temporal and spatial constraints. B cell migration into the brain following viral infection is delayed by several days, which introduces experimental challenges.^1^ First, this delay necessitates a second injection to ensure appropriate temporal exposure of B cells to 4-OHT. Second, to maintain effective local availability of the active compound at the site of infection, 4-OHT must be delivered in a lipophilic formulation that enables slow and sustained diffusion within the targeted brain region. Consequently, recombination is limited to cells in close proximity, potentially failing to target a substantial fraction of B cells infiltrating more distant brain regions.

## Troubleshooting

### Problem 1

During drilling of the skull, a blood vessel (e.g., a vein) may be damaged, leading to substantial bleeding that is difficult to control and may interfere with the procedure.

### Potential solution

• Prepare small pieces of Gelfoam pre-soaked in PBS in advance. In case of bleeding, gently place a piece of the PBS-soaked Gelfoam onto the affected area. This promotes hemostasis and helps to control the bleeding, allowing the procedure to continue once the area is stabilized.

### Problem 2

The prepared mixtures (with or without 4-OHT) may become too viscous, preventing successful loading into the Hamilton syringe.

### Potential solution

• To reduce viscosity during aspiration, warm both the mixture and the Hamilton syringe prior to loading. This helps prevent premature solidification inside the needle or syringe, which can block aspiratio.
• If drawing the mixture into the syringe is unsuccessful, use an insulin syringe (or another syringe with a higher gauge needle) to first take up the mixture. Remove the plunger from the Hamilton syringe and insert the needle of the insulin syringe into the Hamilton syringe barrel. Slowly dispense the mixture into the Hamilton syringe from the top. Carefully reinsert the plunger and verify that the mixture can be smoothly ejected before use.

## Resource availability

### Lead contact

Further information and requests for resources and reagents should be directed to and will be fulfilled by the lead contact, Nicholas S. R. Sanderson (nicholas.sanderson@unibas.ch).

### Technical contact

Technical questions on executing this protocol should be directed to and will be answered by the technical contact, Mika Schneider (mikaschneider@gmx.ch).

### Materials availability

This study did not generate new unique reagents.

### Data and code availability

This study did not generate new data and code.

## Acknowledgments

We are grateful for technical support from Gregor Hutter, from the Microscopy and Flow Cytometry Cores of the Department of Biomedicine of the 10.13039/100008375University of Basel; the Animal Research Station of the University of Basel; and the University of Basel scientific computing center (http://scicore.unibas.ch/). Funding was received from the 10.13039/501100001711Swiss National Science Foundation 169674, 189043, 10001495, and 10000065. The graphical abstract was created with Biorender.com (Created in BioRender. Schneider, M. (2026) https://BioRender.com/wfpx3vw).

## Author contributions

Conceptualization, M.S., N.S.R.S., and T.D.; methodology, M.S., G.P., and N.S.R.S.; investigation, M.S.; writing – original draft, M.S., N.S.R.S., and T.D.; funding acquisition, N.S.R.S. and T.D.; supervision, N.S.R.S. and T.D.

## Declaration of interests

The authors declare no competing interests.
